# The multifunctional solute carrier 3A2 (SLC3A2) confers a poor prognosis in the highly proliferative breast cancer subtypes

**DOI:** 10.1038/s41416-018-0038-5

**Published:** 2018-03-16

**Authors:** Rokaya El Ansari, Madeleine L. Craze, Maria Diez-Rodriguez, Christopher C. Nolan, Ian O. Ellis, Emad A. Rakha, Andrew R. Green

**Affiliations:** 1 Academic Pathology, Division of Cancer and Stem Cells, School of Medicine, Nottingham City Hospital, University of Nottingham, Hucknall Road, Nottingham, NG5 1PB UK; 2 Breast Institute, Nottingham University Hospitals NHS Trust, Hucknall Road, Nottingham, NG5 1PB UK

**Keywords:** Breast cancer, Biomarkers

## Abstract

Breast cancer (BC) is a heterogeneous disease characterised by variant biology, metabolic activity and patient outcome. This study aimed to evaluate the biological and prognostic value of the membrane solute carrier, SLC3A2 in BC with emphasis on the intrinsic molecular subtypes. SLC3A2 was assessed at the genomic level, using METABRIC data (*n* = 1980), and at the proteomic level, using immunohistochemistry on tissue microarray (TMA) sections constructed from a large well-characterised primary BC cohort (*n* = 2500). SLC3A2 expression was correlated with clinicopathological parameters, molecular subtypes and patient outcome. SLC3A2 mRNA and protein expression were strongly correlated with higher tumour grade and poor Nottingham prognostic index (NPI). High expression of SLC3A2 was observed in triple-negative (TN), HER2+ and ER+ high-proliferation subtypes. SLC3A2 mRNA and protein expression were significantly associated with the expression of c-MYC in all BC subtypes (*p* < 0.001). High expression of SLC3A2 protein was associated with poor patient outcome (*p* < 0.001), but only in the ER+ high-proliferation (*p* = 0.01) and TN (*p* = 0.04) subtypes. In multivariate analysis SLC3A2 protein was an independent risk factor for shorter BC-specific survival (*p* < 0.001). SLC3A2 appears to play a role in the aggressive BC subtypes driven by MYC and could act as a potential prognostic marker. Functional assessment is necessary to reveal its potential therapeutic value in the different BC subtypes.

## Introduction

Metabolic reprogramming has been readily accepted as part of the revised hallmarks of cancer where tumour cells are able to modulate their metabolic pathways to support their unremitting proliferation.^1^ Amino-acid transport systems are essential for the growth of cancer cells, not only because they provide amino acids required for protein synthesis but also they activate mammalian target of rapamycin complex 1 (mTORC1), which in turn regulates protein translation and cell growth.^2, 3^ There is also growing evidence that crosstalk can occur among oncogenes and/or tumour suppressor genes and altering the cancer cell metabolism, including the direct regulation of the solute carrier (SLC) family 3 member 2 (SLC3A2) by the oncogene *MYC*.^4^

Recently, membrane transporters have attracted great attention for their crucial roles in cancer proliferation and survival. SLC3A2, also known as CD98hc, is a transmembrane protein, which primarily acts as a chaperone that heterodimerises with a group of amino-acid transporters (e.g., SLC7A5 and SLC7A11) for their functional expression in the plasma membrane.^5, 6^ SLC3A2 also has a biological role in favouring cancer growth, as it associates and regulates the function of β1 integrins and its overexpression leads to amplification of integrin-dependent signals, which involves extracellular matrix remodelling resulting in promoting tumourigenesis and cell proliferation.^7, 8^

SLC3A2 is highly expressed in various cancer types, including gastric cancer,^9^ osteosarcoma,^10^ renal cell carcinoma^11^ and biliary tract cancer.^12^ Previous studies of SLC3A2 in human breast cancer (BC) showed its prognostic significance but in a limited number of cases.^13, 14^ To our knowledge there is no prognostic analysis, which involves the impact of SLC3A2 overexpression in large cohorts, including the different BC molecular subtypes.

In this study, we aimed to assess *SLC3A2* gene copy number (CN) and mRNA expression alongside protein expression in large and well-characterised annotated cohorts of BC to determine its clinicopathological and prognostic value with emphasis on the different molecular classes.

## Materials and methods

### *SLC3A2* genomic profiling

A cohort of 1980 invasive BC in the Molecular Taxonomy of Breast Cancer International Consortium (METABRIC)^15^ was used to evaluate *SLC3A2* gene CN aberrations and gene expression. In the METABRIC study, DNA/RNA was isolated from fresh frozen samples and transcriptional profiling was obtained using the Illumina HT-12v3 platforms. Data were pre-processed and normalised as described previously.^15^ In this cohort, patients who were oestrogen receptor-positive (ER+) and/or lymph node (LN)-negative did not receive adjuvant chemotherapy, whereas ER− and LN+ patients were offered adjuvant chemotherapy. None of the patients were treated with anti-HER2-targeted therapy. Dichotomisation of *SLC3A2* mRNA expression was determined using the median value as the cutoff point. The association between the *SLC3A2* mRNA expression and clinicopathological parameters, molecular subtypes and patient outcome was investigated.

The online dataset, Breast Cancer Gene Expression Miner v4.0 (http://bcgenex.centregauducheau.fr), was used for external validation of SLC3A2 mRNA expression.

### SLC3A2 protein expression

Immunhisotchemistry for SLC3A2 was performed using a well-characterised cohort of early-stage primary operable invasive BC patients aged ≤70 years. Patients presented at Nottingham City Hospital between 1989 and 2006. Patients were managed based on a uniform protocol. Clinical history, tumour characteristics, information on therapy and outcomes are prospectively maintained. Outcome data included development and time to distant metastasis (DM) and BC-specific survival (BCSS).

The clinicopathological parameters for the Nottingham and METABRIC series are summarised in (Supplementary Table 1).

### Western blotting

**Fig. 1:**
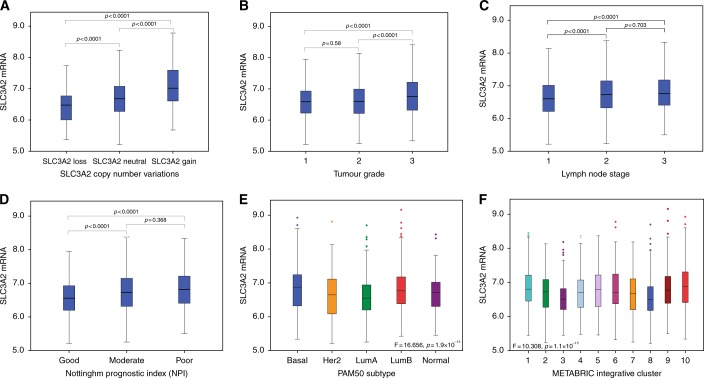
Western blotting results for **a** SLC3A2 expression in MDA-MB-231 breast cancer cell lysates and SLC3A2 protein expression in invasive breast cancer cores. **b** Positive IHC expression, **c** negative IHC expression

The antibody specificity of anti-SLC3A2 (HPA017980, Sigma-Aldrich, UK) was validated using western blotting in MDA-MB-231 BC lysate (American Type Culture Collection; Rockville, MD, USA) as previously described.^16^ A single band for SLC3A2 was visualised at the correct predicted size (80 kDa; Fig. 1a).

### Tissue arrays and immunohistochemistry

Tumour samples, 0.6 mm cores, were arrayed as previously described.^17^ Immunohistochemical staining was performed on 4 μm tissue micoarray (TMA) sections using Novolink polymer detection system (Leica Biosystems, RE7150-K) as previously described.^16^

Stained TMA sections were scanned using high-resolution digital images (NanoZoomer; Hamamatsu Photonics, Welwyn Garden City, UK), at ×20 magnification. Evaluation of staining for SLC3A2 was based on a semiquantitative assessment of cores’ digital images using a modified histochemical score (*H*-score), which includes an assessment of both the intensity and the percentage of stained cells.^18^ Staining intensity was assessed as follows 0, negative; 1, weak; 2, medium; 3, strong and the percentage of the positively stained tumour cells was estimated subjectively. The final *H*-score was calculated multiplying the percentage of positive cells (0–100) by the intensity (0–3), producing a total range of 0–300. Dichotomisation of protein expression was determined using the median value as the cutoff point.

Immunhistochemical staining and dichotomisation of the other biomarkers included in this study were as per previous publications^17, 19–27^ (Supplementary table 2). ER and PgR positivity was defined as ≥1% staining. Immunoreactivity of HER2 was scored using standard HercepTest guidelines (Dako). Chromogenic in situ hybridisation (CISH) was used to quantify *HER2* gene amplification in borderline cases using the HER2 FISH pharmDx™ plus HER2 CISH pharmDx™ kit (Dako) and was assessed according to the American Society of Clinical Oncology guidelines. BC molecular subtypes were defined, based on tumour immunohistochemical profile and the Elston-Ellis^28^ mitotic score as: ER+/HER2− low proliferation (mitotic score 1) and ER+/HER2− high proliferation (mitotic score 2 and 3); HER2-positive class: HER2+ regardless of ER status; triple-negative (TN): ER−, PgR− and HER2−.^29^

### Statistical analysis

Statistical analysis was performed using SPSS 22.0 statistical software (SPSS Inc., Chicago, IL, USA). Spearman’s correlation coefficient was carried out to examine the association between continuous variables. The Chi-square test was performed for inter-relationships between categorical variables. Survival curves were analysed by Kaplan–Meier with log rank test using BC-specific death censoring cases who were lost to follow-up or died of other causes. Cox’s proportional hazard method was performed for multivariate analysis to identify the independent prognostic/predictive factors and the proportional hazard assumption was tested with Schoenfeld residuals test to avoid violation of the assumption. The statistical test for heterogeneity was applied to assess the difference between the subtypes. *p*-values were adjusted using Bonferroni correction for multiple testing, whenever applicable. A *p*-value < 0.05 was considered significant. The study end points were 5-year BCSS or DM-free survival (DMFS).

This study was approved by the Nottingham Research Ethics Committee 2 under the title ‘Development of a molecular genetic classification of breast cancer’. All samples from Nottingham used in this study were pseudo-anonymised and collected prior to 2006 and therefore under the Human Tissue Act informed patient consent was not needed. Release of data was also pseudo-anonymised as per Human Tissue Act regulations.

## Results

### SLC3A2 genomic profiling in BC

**Fig. 2:**
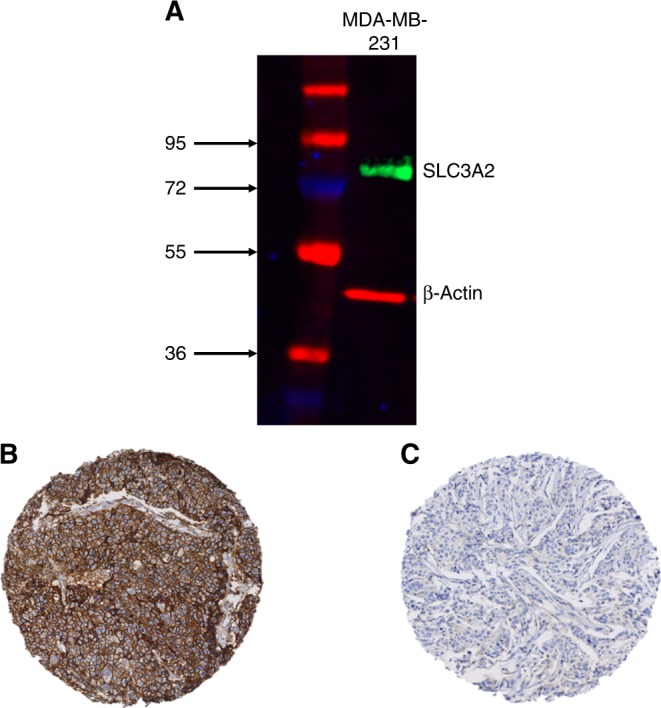
*SLC3A2* expression and its association with copy number aberrations, clinicopathological parameters and molecular subtypes: **a** *SLC3A2* and gene copy number variations; **b** *SLC3A2* and tumour grade; **c** *SLC3A2* and lymph node stage; **d** *SLC3A2* and NPI; **e** *SLC3A2* and PAM50 subtypes; **f** *SLC3A2* and METABRIC integrative clusters

High SLC3A2 mRNA expression was observed in 961/1858 (52%) of the METABRIC BC cases. In all, 90 (4.5%) of cases showed *SLC3A2* CN gain, whereas 109 (5.5%) showed a CN loss. A significant association was observed between *SLC3A2* CN variation and *SLC3A2* mRNA expression (*p* < 0.001, Fig. 2a). There was a positive association between *SLC3A2* CN gain and its regulator, *MYC*, gain (*p* < 0.001, Supplementary Table 2).

High *SLC3A2* mRNA expression was significantly associated with higher tumour grade (Fig. 2b, *p* < 0.001), positive nodal metastasis (Fig. 1c, *p* < 0.001) and poor Nottingham Prognostic Index (NPI; Fig. 2d, *p* < 0.001). These associations were confirmed using the Breast Cancer Gene-Expression Miner v4.0 (Supplementary Figure 1A, 1B).

The correlation of *SLC3A2* mRNA with other relevant genes were investigated using the METABRIC dataset (Supplementary Table 4). The genes were selected based on previous publications, being either regulatory genes or those that share or support the SLC3A2 biological function focussing primarily on the amino-acid transport system. There was a relationship between *SLC3A2* and the regulatory genes, *ATF4* (*p* = 0.02) and *MYC* with the latter showing significant correlation across all BC subtypes (*p* < 0.001). High *SLC3A2* mRNA expression was significantly associated with its heterodimers, the glutamine exchanger, *SLC7A5* and, the glutamate transporter, *SLC7A11* (all *p* = 0.002). Nevertheless, the majority of other glutamine transporters were negatively correlated with the *SLC3A2* expression. A similar association was also observed with glutaminase (GLS) enzyme, which mediates the conversion of glutamine to glutamate (*p* < 0.001). High *SLC3A2* mRNA expression was associated with those tumours where there were *TP53* mutations (*p* < 0.001, Table 1).

**Table 1:** Expression of SLC3A2 in breast cancer and the expression of other molecular biomarkers

	SLC3A2 (mRNA)				SLC3A2 (protein)			
	Low*n* (%)	High*n* (%)	*χ*^2^(*p*-value)	Adjusted *p*-value	Low*n* (%)	High*n* (%)	*χ*^2^(*p*-value)	Adjusted *p*-value
ER								
Negative	182 (40.1)	272 (59.9)	16.138		208 (38.1)	338 (61.9)	44.97	
Positive	715 (50.9)	689 (49.1)	(**0.0003**)	**0.0009**	1051 (54.3)	883 (45.7)	(1.9 × 10^−11^)	**<0.0001**
PR								
Negative	390 (43.6)	504 (56.4)	14.943		708 (46.4)	818 (53.6)	28.5	
Positive	507 (52.6)	457 (47.4)	(**0.001**)	**0.002**	466 (58.0)	337 (42.0)	(9.3 × 10^−8^)	**<0.0001**
HER2								
Negative	799 (49.2)	826 (50.8)	4.125		1090 (51.9)	1009 (48.1)	16.14	
Positive	98 (42.1)	135 (59.9)	(**0.04**)	0.08	94 (38.4)	151 (61.6)	(0.00005)	**<0.0001**
Triple negative								
No	786 (50.7)	765 (49.3)	21.64		1082 (53.0)	961 (47.0)	23.61	
Yes	111 (36.2)	196 (63.8)	(**0.000003**)	**<0.0001**	156 (39.6)	238 (60.4)	(0.000001)	**0.0001**
TP53 mutations								
Wild type	280 (42.1)	385 (57.9)	37.41		N/A			
Mutation	27 (28.7)	67 (71.3)	(**3.7** × **10**^**−8**^)	**<0.0001**				
p53 protein								
Negative	N/A				499 (76.7)	152 (23.3)	40.299	
Positive					146 (55.5)	269 (29.4)	(2.1 × 10^−10^)	**<0.0001**

*P* values in bold means statistically significant

### SLC3A2 protein expression in BC

SLC3A2 protein expression was observed, predominantly in the membrane of invasive BC cells, with expression levels varying from absent to high (Fig. 1b, c). Positive SLC3A2 protein expression (>15 *H*-score) was observed in 50% of the cases.

Table 2 summarises the observed associations with high SLC3A2 protein expression, including larger tumour size (*p* = 0.006), high tumour grade (*p* < 0.001) and poor NPI (*p* < 0.001). In addition, high SLC3A2 protein was associated with medullary-like tumours (*p* < 0.001). Regarding BC metastatic sites, high SLC3A2 protein levels were associated with the development of distant metastases to the brain (*p* < 0.001) while there was no association with developing DM to the bone or lung.

**Table 2:** Clinicopathological associations of SLC3A2 expression in breast cancer

	SLC3A2 protein			
	Low*n* (%)	High*n* (%)	*χ*2(*p*-value)	Adjusted *p*-value
Tumour size				
≥2.0 cm	526 (47.7)	576 (52.3)	7.49	
<2.0 cm	736 (53.3)	646 (46.7)	(0.006)	**0.03**
Tumour grade				
1	252 (62.7)	150 (37.3)		
2	543 (56.9)	412 (43.1)	76.34	
3	466 (41.4)	659 (58.6)	(2.6 × 10^−17^)	**<0.0001**
Lymph node stage				
1	794 (51.2)	756 (48.8)		
2	350 (50.1)	349 (49.9)	0.292	
3	115 (50.2)	114 (49.8)	(0.86)	1.72
Nottingham Prognostic Index (NPI)				
Good	481 (58.9)	337 (41.1)		
Moderate	611 (48.2)	657 (51.8)	36.37	
Poor	167 (42.5)	226 (57.5)	(8.01 × 10^−7^)	**<0.0001**
IHC subtypes				
ER+/HER2− low proliferation	748 (58.0)	542 (42.0)		
ER+/HER2− high proliferation	156 (42.9	208 (57.1)	66.58	
Triple negative	153 (39.5)	234 (60.5)	(2.3 × 10^−14^)	**<0.0001**
Histological type				
Ductal (including mixed)	1039 (49.5)	1060 (50.5)		
Lobular	141 (62.7)	84 (37.3)		
Medullary	9 (25.7)	26 (74.3)	29.73	
Miscellanous	9 (50.0)	9 (50.0)	(0.0002)	**0.001**
Special type	59 (57.3)	44 (42.7)		
HER2+	94 (38.4)	151 (61.6)		
Site of distant metastasis				
Brain				
No	648 (72.6)	245 (27.4)	17.08	
Yes	25 (46.3)	29 (53.7)	(0.00003)	**0.0002**
Viscera				
No	649 (70.4)	273 (29.6)	5.065	
Yes	26 (89.7)	3 (10.3)	(0.02)	0.1
Bone				
No	529 (70.9)	217 (29.1)	0.041	
Yes	144 (71.6)	57 (28.4)	(0.839)	2.51
Lung				
No	618 (71.7)	244 (28.3)	1.837	
Yes	55 (64.7)	30 (35.3)	(0.175)	0.7

*P* values in bold means statistically significant

SLC3A2 protein was significantly expressed with high Ki67 and c-MYC expression (*p* < 0.001, Supplementary Table 5). SLC7A5, SLC1A5, GLS and PIK3CA were significantly expressed in breast tumours with high expression of SLC3A2 (*p* < 0.001), while the low expression was associated with high levels of p-mTORC1 (*p* < 0.001, Supplementary Table 5). Moreover, high SLC3A2 protein was positively associated with high nuclear p53 protein expression (*p* < 0.001, Table 1).

### SLC3A2 expression in molecular BC intrinsic subtypes

High expression of *SLC3A2* mRNA was significantly associated with hormone receptor-negative (ER− and PR−) tumours (*p* ≤ 0.001, Table 1) but not with HER2+ BC. These results were in concordance with the Breast Cancer Gene-Expression Miner v4.0 (Supplementary Figure 1C-F). Similarly, SLC3A2 protein expression was associated with negative hormone status and HER2+ tumours (all *p* ≤ 0.001, Table 1) and it was highly expressed in TN compared with non-TN tumours (*p* < 0.001, Table 1).

When comparing the levels of *SLC3A2* CN and mRNA expression in the intrinsic (PAM50) subtype,^30^ high mRNA expression was observed in basal-like, luminal B and HER2+ tumours (Fig. 2e, *p* < 0.001), whereas *SLC3A2* CN gain was primarily observed in luminal B subtype and to lesser extent in HER2+ and triple negative breast cancer  (TNBC) (*p* < 0.001, Supplementary Table 3). In the METABRIC Integrative Clusters, high *SLC3A2* mRNA expression was associated with clusters 1 (luminal B subgroup), 5 (ERBB2-amplified) and 10 (TN/basal-like) with proportions 63%, 61% and 65%, respectively (*p* < 0.001, Fig. 2f). Association of *SLC3A2* mRNA with the molecular subtypes was confirmed using the Breast Cancer Gene-Expression Miner v4.0 (Supplementary Figure 1G).

Expression of SLC3A2 protein in the defined molecular subtypes showed a lower expression in the ER+ low-proliferation tumours compared with the other subtypes (*p* < 0.001, Table 2).

### SLC3A2 expression and patient outcome

**Fig. 3:**
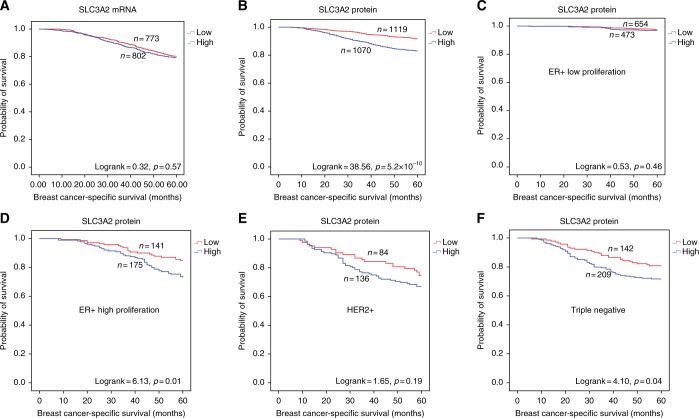
SLC3A2 and breast cancer patient outcome. **a** *SLC3A2* vs BCSS in all cases, **b** SLC3A2 vs BCSS in all cases, **c** SLC3A2 vs BCSS of ER+ low-proliferation tumours, **d** SLC3A2 vs BCSS of ER+ high-proliferation tumours, **e** SLC3A2 vs BCSS in HER2+ tumours, **f** SLC3A2 vs BCSS in triple-negative tumours

High SLC3A2 protein expression, but not mRNA, was associated with poor outcome in terms of shorter BCSS (*p* < 0.001, Fig. 3a, b). When investigating within the molecular classes, high expression of SLC3A2 protein was predictive of shorter BCSS in ER+ high-proliferation class (*p* = 0.01, Fig. 3d), and TN tumours (*p* = 0.04, Fig. 3f). There was no association between SLC3A2 protein and outcome in HER2+ (Fig. 3e) and ER+ low-proliferation tumours (Fig. 3c).

High SLC3A2 protein expression was associated with shorter DMFS (*p* < 0.001, Supplementary Figure 2A) and this was only observed in ER+ high-proliferation and TN tumours (*p* = 0.04, Supplementary Figure 2C, 2E) but not with other two subtypes (Supplementary Figure 2B, 2D).

In multivariate Cox regression analysis, SLC3A2 protein was a predictor of shorter BCSS (*p* < 0.001, Table 3) independent of tumour size, grade and stage. The same significant result was remained in the ER+ high-proliferation and TN tumours (*p* = 0.01, Supplementary table 6) when different subtypes were considered. However, heterogeneity test revealed no evidence of a difference in the observed effects for the three aggressive subtypes, ER+ high proliferation, HER2+ and TN tumours, after adjusting the confounding variables (*p* = 0.91 and 0.90 in BCSS and DMFS, respectively; data not shown).

**Table 3:** Univariate and multivariate analysis of prognostic variables and SLC3A2 expression in relation to BCSS

		SLC3A2 protein			
		Univariate		Multivariate	
Variable		Hazard ratio (95% CI)	*p*-value	Hazard ratio (95% CI)	*p*-value
SLC3A2	Low vs high	2.17 (1.69–2.79)	**1.4 **×** 10**^**−9**^	1.83 (1.42–2.36)	**0.000003**
Size	<2 cm vs ≥2 cm	2.89 (2.37–3.53)	**1.1** × **10**^**−25**^	1.55 (1.19–2.01)	**0.001**
Grade	G1 vs G2 and 3	4.39 (3.55–5.43)	**2.5** × **10**^**−42**^	4.04 (3.00–5.43)	**2.8 **×** 10**^**−20**^
Lymph node stage	N1 vs N2 and 3	2.49 (2.21–2.81)	**2.2** × **10**^**−50**^	2.11 (1.81–2.47)	**4.5 **×** 10**^**−21**^

*P* values in bold means statistically significant

## Discussion

BC is a heterogeneous disease^31^ and the high level of diversity among the various subtypes is reflected on the clinical behaviour, response to therapy and patient outcome. In addition, different subtypes exhibit a disparity in their metabolic pathways and nutritional needs. ER+/luminal tumours are the most common BC subtypes,^32, 33^ which are also different it terms of disease prognosis and mortality rates.^32^

SLC proteins are related to tumourigenesis and drug resistance in cancer cells^34^ and SLC3A2 is characterised by its dual effect to promote cancer cell growth and survival. Besides its role in regulating the function of amino-acid transporter systems, it modulates integrin-induced signal transduction, which derives malignant tumour cells’ behaviour, including cell spreading and migration.^35^

The present study involved a large BC cohort to reveal the significant association between the high SLC3A2 protein expression and the poor prognostic clinicopathological parameters. Furthermore, high SLC3A2 expression was significantly associated with proliferation. This supports the results of previous studies, which reported that these, SLC3A2 and Ki67, are significantly correlated in non-small-cell lung cancer^36^ and hypo-pharyngeal squamous cell carcinoma,^37^ confirming that SLC3A2 is critical for proliferation in cancer cells.

Regarding the ER+ BC subtypes, SLC3A2 expression was lower in ER+ tumours that have low proliferation compared with the highly proliferative ER+ tumours, and it was associated with poor patient outcome in the latter class only. This is doubtlessly attributable to their aggressive character as well as their heavier nutrient requirements for cell survival and proliferation.

SLC3A2 protein was also highly expressed in TNBC and HER2+, in concordance with Furuya et al.^14^ However, the significant association between SLC3A2 protein expression and patient outcome was only restricted to TNBC. In this regard, it has been shown that over-expression of SLC3A2 is actively involved in the proliferation of vascular smooth muscle cells and is necessary for efficient angiogenesis.^38, 39^ In this study, ER+ high-proliferation and TN tumours showed the most significant positive correlation between the mRNA expression of SLC3A2 and vascular endothelial growth factor B, which maintains the continuity of angiogenesis and thus implicated for the metastatic process.^40^ Although the association between HER2+ tumours and patient outcome was not nominally significant, there was no difference in the effect of this subtype and the other aggressive tumour types.

Generally, the association with patient outcome was observed at the protein, but not the mRNA, level. This can be attributed to the post-translational modification, *N*-glycosylation, of the SLC3A2 protein, which is required to make this protein functioning as it is renowned that the glycosylated SLC3A2 (~80 kDa) is necessary to form the heterodimeric complex, which further assist the amino-acid transport function.

Previous studies have showed regulation of SLC3A2 by other proteins, including the tumour oncogene *c-Myc*.^4^ In the current study, the relationship between SLC3A2 and other regulatory proteins in both mRNA and protein expression was investigated. A positive relationship was observed between SLC3A2 and c-Myc, at both protein and mRNA levels and this correlation was observed in all BC subtypes when tested at the mRNA level. However, it was only significant with the ER+ high-proliferation and TN tumours (p = 0.006 and 0.002), respectively, when investigated at the protein level (data not shown).

The heavy chain of SLC3A2 forms a disulphide bond with the light chain of a group of amino-acid transporters mediating their functions in the plasma membrane. The most prominent is the glutamine exchanger (SLC7A5), which imports the essential amino acids to cancer cells in exchange for intracellular glutamine, the procedure that subsequently activates mTORC1.^2, 3^ Another protein that heterodimerises with SLC3A2 is the cysteine-glutamate transporter (SLC7A11), which potentiates the cellular antioxidant machinery through assisting the glutathione synthesis.^41^ This study revealed the depth of the positive correlation between these amino-acid transporters, specifically for SLC7A5, which remained significant in almost all subtypes, apart from in luminal A tumours. Hence, we investigated the association of SLC3A2 with the downstream signal, mTORC1. However, high SLC3A2 protein expression was associated with lower expression of the mTORC1 phosphorylated at ser (2448), which was included in this study, and this attributed to what was confirmed by Cheng et al. that phosphorylation of mTORC1 at ser (2448), which is stimulated by growth factors, was mutually exclusive with mTORC1 phosphorylated at thr (2446), which is regulated by amino acids.^42^

This study further investigated the association of SLC3A2 expression with other glutamine transporters, which provide the substrate, glutamine, required to operate the SLC3A2-SLC7A5 heterodimeric complex. GLS, which converts glutamine to glutamate, is the substrate needed for SC3A2-SLC7A11 function. Although the mRNA showed a negative correlation with most glutamine transporters and GLS, the high SLC3A2 protein expression was associated with the higher levels of the glutamine transporter (SLC1A5), SLC7A5 and GLS, indicating a system of functional coupling between these biomarkers at the protein level.

The role of the tumour microenvironment is well-known with respect to disease development and progression, and SLC3A2 appears to have a role in this, as the SLC3A2 heavy chain binds to the cytoplasmic tail of integrin β1, which in turn mediates extracellular matrix remodelling that controls cell spreading, survival and growth.^43, 44^ The SLC3A2 interaction with integrin is well studied in renal cancer cell^35^ and the current study also confirmed the positive correlation between gene expressions in all BC subtypes.

A recent study reported that SLC3A2 influences osteosarcoma growth through the PI3K/AKt signalling pathway^10^ and this could be the case in BC as demonstrated by our finding that high levels of SLC3A2 protein is positively associated with PIK3CA expression. Therefore, it appears that all functions of SLC3A2 in BC were associated with poor prognosis and would not be in favour of patients with aggressive subtypes.

Targeting SLC3A2 efficiently decreases colony formation of osteosarcoma cells^10^ and affects renal cancer cell growth in vivo.^35^ The consequences of blocking SLC3A2 is therefore warranted in the aggressive highly proliferative BC subtypes.

## Conclusion

This study revealed that SLC3A2 was associated with poor prognostic characteristics and poor survival outcome. Over-expression of SLC3A2 appears to play a role in the proliferation and progression of the highly proliferative ER+, HER2+ and TN subtypes of BC, thus it could act as a potential prognostic marker and therapeutic target. Functional assessment is necessary to reveal the specific role played by this membrane protein in the highly proliferative more aggressive BC subclasses.

## Electronic supplementary material

Supplementary tables and figures
