# Supplementary material for: The multifunctional solute carrier 3A2 (SLC3A2) confers a poor prognosis in the highly proliferative breast cancer subtypes
Source: Br J Cancer. 2018 Mar 16;118(8):1115–22. doi: 10.1038/s41416-018-0038-5 (PMC5931111; doi:10.1038/s41416-018-0038-5)
Supplement: Supplementary file 1 — Supplementary tables and figures [file 41416_2018_38_MOESM1_ESM.docx]

**Supplementary Table 1.** Clinicopathological parameters of the METABRIC and Nottingham breast cancer series.

|  | **METABRIC series**  **n (%)** | **Nottingham TMA series**  **n (%)** |
| --- | --- | --- |
| **Age** |  |  |
| ≥ 50 years | 1426 (78.6) | 1784 (67.4) |
| ˂ 50 years | 424 (21.4) | 864 (32.6) |
| **Tumour size** |  |  |
| ≥ 2cm | 1337 (68.2) | 1176 (44.4) |
| ˂2cm | 623 (31.8) | 1471 (55.6) |
| **Grade** |  |  |
| 1 | 169 (9.0) | 421 (15.9) |
| 2 | 770 (40.7) | 988 (37.4) |
| 3 | 952 (50.3) | 1235 (46.7) |
| **Tumour type** |  |  |
| Ductal (including mixed) | 1545 (83.6) | 2257 (85.3) |
| Lobular | 148 (8.0) | 221 (8.4) |
| Medullary-like | 32 (1.7) | 39 (1.5) |
| Miscellaneous | 12 (0.6) | 16 (0.6) |
| Special type | 113 (6.1) | 113 (4.2) |
| **Vascular Invasion** |  |  |
| Definite | Not available | 833 (31.5) |
| Negative/Probable |  | 1808 (68.5) |
| **Lymph Node Stage** | | |
| 1 | 1035 (52.5) | 674 (61.0) |
| 2 | 623 (31.5) | 341 (30.8) |
| 3 | 315 (16.0) | 91 (8.2) |
| **Follow-up Status** |  |  |
| Alive | 1070 (55.7) | 1679 (63.4) |
| Died from Breast Cancer | 505 (26.3) | 647 (24.4) |
| Died from other causes | 345 (18.0) | 321 (12.2) |
| **ER** |  |  |
| Negative | 472 (23.8) | 570 (21.6) |
| Positive | 1508 (76.2) | 2067 (78.4) |
| **PgR** |  |  |
| Negative | 938 (47.4) | 1047 (41.4) |
| Positive | 1042 (52.6) | 1483 (58.6) |
| **HER2** |  |  |
| Negative | 1734 (87.5) | 2297 (88.5) |
| Positive | 246 (12.5) | 298(11.5) |

| **Marker** | Cut-off |
| --- | --- |
| ER | ≥1% |
| PgR | ≥1% |
| HER2 | 3+ and 2+ with amplification |
| Ki67 | ≥10% |
| c-MYC | >35 H-score |
| p53 | >10% |
| PIK3CA | >100 H-score |
| p-mTORC1 | >35 H-score |
| GLS | >120 H-score |
| SLC1A5 | >45 H-score |
| SLC7A5 | >15 H-score |

**Supplementary Table 2:** Biomarkers - cut-offs used in the study

**Supplementary Table 3**: Copy Number Aberrations of *SLC3A2* in breast cancer and their associations with MYC and molecular subtypes

|  | ***SLC3A2* Gain** | | |
| --- | --- | --- | --- |
|  | **No** | **Yes** | **χ2 (p-value)** |
| ***MYC* Gain** |  |  |  |
| No | 1205(97.0) | 37 (3.0) | 18.843 (**0.00001**) |
| Yes | 685 (92.8) | 53 (7.2) |  |
| **Molecular subtypes** |  |  |  |
| Luminal A | 703 (97.9) | 15 (2.1) | 30.912 (**0.000003)** |
| Luminal B | 445 (91.2) | 43(8.8) |  |
| Basal | 315 (95.7) | 14 (4.3) |  |
| HER2 | 229 (95.4) | 11 (4.6) |  |
| Normal-like | 1884 (95.4) | 7 (3.5) |  |

**Supplementary Table 4**: Correlation of *SLC3A2* mRNA expression with the expression of other genes

|  | ***SLC3A2* mRNA** | | | | | | | | | | | | | | | |
| --- | --- | --- | --- | --- | --- | --- | --- | --- | --- | --- | --- | --- | --- | --- | --- | --- |
|  | **All cases**  (n=1,980) | | | **Luminal A**  (n=368) | | | | **Luminal B**  (n=367) | | | | | **HER2+**  (n=110) | | **Triple negative**  (n=150) | |
| **Correlation Coefficient**  **(p-value)** | | | | | | | | | | **Adjusted p-value** | | | | | | |
| **Regulatory and other associated genes** | | | | | | | | | | | | | | | | |
| *MYC* | 0.390  (4.7x10^-73^) | | **<0.0001** | | 0.388  (3.1x10^-27^) | | **<0.0001** | | 0.348  (2.4x10^-15^) | | | **<0.0001** | 0.502  (1.04x10^-16^) | **<0.0001** | 0.301  (2.5x10^-8^) | **<0.0001** |
| *mTOR* | -0.272  (5.2x10^-35^) | | **<0.0001** | | -0.326  (2.7x10^-19^) | | **<0.0001** | | -0.128  (0.005) | | | 0.07 | -0.221  (0.001) | **0.01** | -0.349  (7.0x10^-11^) | **<0.0001** |
| *VEGFB* | 0.149  (2.8x10^-11^) | | **<0.0001** | | 0.129  (0.001) | | **0.01** | | 0.213  (0.000002) | | | **<0.0001** | 0.134  (0.03) | 0.27 | 0.310  (9.2x10^-9^) | **<0.0001** |
| *ITGB1* | 0.459  (1.3x10^-103^) | | **<0.0001** | | 0.458  (1.7x10^-38^) | | **<0.0001** | | 0.314  (1.3x10^-12^) | | | **<0.0001** | 0.676  (1.9x10^-33^) | **<0.0001** | 0.443  (3.2x10^-17^) | **<0.0001** |
| *ATF4* | 0.065  (0.004) | | **0.02** | | -0.018  (0.626) | | 2.50 | | -0.016  (0.720) | | | 4.32 | 0.160  (0.102) | 0.714 | 0.169  (0.002) | **0.02** |
| **Glutamine metabolism** | | | | | | | | | | | | | | | | |
| *GLS* | -0.127  (1.4x10^-8^) | **<0.0001** | | -0.150  (0.00005) | | **0.0006** | | -0.041  (0.365) | | | 3.228 | | 0.160  (0.01) | 0.10 | 0.164 (0.003) | **0.03** |
| **Glutamine/Glutamate transporters** | | | | | | | | | | | | | | | | |
| *SLC7A5* | 0.172  (1.1x10^-14^) | **<0.0001** | | 0.067  (0.072) | | 0.49 | | 0.193  (0.00001) | | | **0.0002** | | 0.184  (0.004) | **0.04** | 0.158  (0.004) | **0.04** |
| *SLC7A11* | 0.081  (0.0003) | **0.002** | | 0.031  (0.409) | | 2.04 | | 0.107  (0.01) | | | 0.14 | | 0.036  (0.574) | 1.72 | 0.138  (0.01) | 0.10 |
| *SLC1A5* | -0.098  (0.00001) | **0.0001** | | -0.153  (0.00003) | | **0.0004** | | -0.054  (0.236) | | | 2.36 | | -0.228  (0.0003) | **0.004** | -0.135  (0.016) | 0.08 |
| *SLC6A19* | 0.041  (0.07) | 0.28 | | 0.073  (0.051) | | 0.40 | | 0.002  (0.967) | | | 2.90 | | 0.022  (0.732) | 1.46 | 0.123  (0.025) | 0.14 |
| *SLC7A6* | -0.211  (2.1x10^-21^) | **<0.0001** | | -0.285  (7.1x10^-15^) | | **<0.0001** | | -0.097  (0.03) | | | 0.39 | | -0.339  (7.0x10^-8^) | **<0.0001** | -0.320  (2.9x10^-9^) | **<0.0001** |
| *SLC7A7* | 0.191  (1.1x10^-17^) | **<0.0001** | | 0.196  (1.2x10^-7^) | | **<0.0001** | | 0.081  (0.075) | | | 0.84 | | 0.235  (0.0002) | **0.003** | 0.137  (0.01) | 0.09 |
| *SLC7A8* | -0.078  (0.001) | **0.005** | | -0.004  (0.915) | | 1.83 | | 0.072  (0.110) | | | 1.21 | | -0.194  (0.003) | **0.03** | -0.072  (0.194) | 0.78 |
| *SLC7A9* | -0.155  (3.9x10^-12^) | **<0.0001** | | -0.186  (4.8x10^-7^) | | **<0.0001** | | -0.037  (0.410) | | | 3.28 | | -0.197  (0.002) | **0.02** | -0.103  (0.063) | 0.36 |
| *SLC38A1* | -0.101  (0.000006) | **0.0001** | | -0.080  (0.03) | | 0.27 | | 0.001  (0.978) | | | 1.95 | | -0.037  (0.568) | 2.27 | -0.222  (0.00004) | **0.0006** |
| *SLC38A2* | -0.223  (7.7x10^-24^) | **<0.0001** | | -0.231  (3.9x10^-10^) | | **<0.0001** | | -0.188  (0.00002) | | | **0.0003** | | -0.283  (0.000009) | **0.0002** | -0.199  (0.0002) | **0.002** |
| *SLC38A3* | -0.064  (0.004) | **0.02** | | -0.114  (0.002) | | **0.02** | | -0.033  (0.464) | | | 3.24 | | -0.113  (0.08) | 0.64 | -0.082  (0.139) | 0.69 |
| *SLC38A5* | -0.016  (0.472) | 0.94 | | -0.016  (0.673) | | 2.02 | | 0.004  (0.933) | | | 3.73 | | -0.101  (0.118) | 0.71 | -0.059  (0.290) | 0.87 |
| *SLC38A7* | -0.297  (1.4x10^-41^) | **<0.0001** | | -0.354  **(1.2x10^-22^)** | | <0.0001 | | -0.143  (0.002) | | | **0.03** | | -0.578  (8.9x10^-23^) | **<0.0001** | -0.318  (3.7x10^-9^) | **<0.0001** |
| *SLC38A8* | 0.034  (0.134) | 0.40 | | 0.37  (0.322) | | 1.93 | | -0.011  (0.812) | | | 0.40 | | -0.041  (0.526) | 2.63 | 0.034  (0.538) | 0.11 |

**Supplementary Table 5**: Association between SLC3A2 protein expression and other biomarkers.

|  | **All cases** | | |
| --- | --- | --- | --- |
|  | **Low, n (%)** | **High, n (%)** | **χ2**  **(p-value)** |
| **c-MYC** |  |  |  |
| Negative | 341 (51.7) | 318 (48.3) | 20.73  (**0.000005**) |
| Positive | 35 (29.2) | 85 (70.8) |  |
| **Ki67** |  |  |  |
| Negative | 158 (61.7) | 98 (38.3) | 18.28  (**0.00001**) |
| Positive | 238 (45.4) | 286 (54.6) |  |
| **P-mTORC1** |  |  |  |
| Negative | 182 (42.6) | 245 (57.4) | 19.35  (**0.00001**) |
| Positive | 183(59.0) | 127 (41.0) |  |
| **PIK3CA** |  |  |  |
| Negative | 109 (61.1) | 68 (38.4) | 13.67  (**0.0002**) |
| Positive | 248 (45.6) | 296 (54.4) |  |
| **SLC1A5** |  |  |  |
| Negative | 275 (68.4) | 127 (31.6) | 92.92  (**5.4x10^-22^**) |
| Positive | 201 (36.7) | 346 (63.3) |  |
| **SLC7A5** |  |  |  |
| Negative | 447 (61.2) | 283 (38.8) | 206.85  (**6.6x10^-47^**) |
| Positive | 2 (1.1) | 176 (98.9) |  |
| **GLS** |  |  |  |
| Negative | 274 (58.7) | 193 (41.3) | 62.18  **(3.1x10^-15^)** |
| Positive | 101 (30.4) | 231 (69.6) |  |

**Supplementary table 6**: multivariate analysis of prognostic variables and SLC3A2 expression, in relation to BCSS, in different BC subtypes.

| **SLC3A2 protein** | | | | | | | | |
| --- | --- | --- | --- | --- | --- | --- | --- | --- |
|  | **ER-low proliferation** | | **ER-High proliferation** | | **Triple Negative** | | **HER2+** | |
| **Variable** | **Hazard ratio**  **(95% CI)** | **p-value** | **Hazard ratio**  **(95% CI)** | **p-value** | **Hazard ratio**  **(95% CI)** | **p-value** | **Hazard ratio**  **(95% CI)** | **p-value** |
| **SLC3A2** | 1.36 (0.67-2.76) | 0.392 | 1.86 (1.11-3.12) | **0.01** | 1.77 (1.11-2.81) | **0.01** | 1.58 (0.94-2.69) | 0.08 |
| **Size** | 1.53 (0.69-3.34) | 0.291 | 1.73 (0.97-3.07) | 0.06 | 1.40 (0.88-2.23) | 0.153 | 1.71 (0.99-2.94) | 0.05 |
| **Grade** | 1.27 (0.68-2.38) | 0.449 | 2.55 (0.35-18.41) | 0.354 | 2.23 (0.84-5.90) | 0.107 | 1.75 (0.87-3.54) | 0.12 |
| **Stage** | 3.55 (2.15-5.88) | **8.2x10^-7^** | 1.57 (1.14-2.15) | **0.005** | 2.29 (1.73-3.05) | **7.9x10^-9^** | 2.24 (1.63-3.10) | **7.8x10^-7^** |


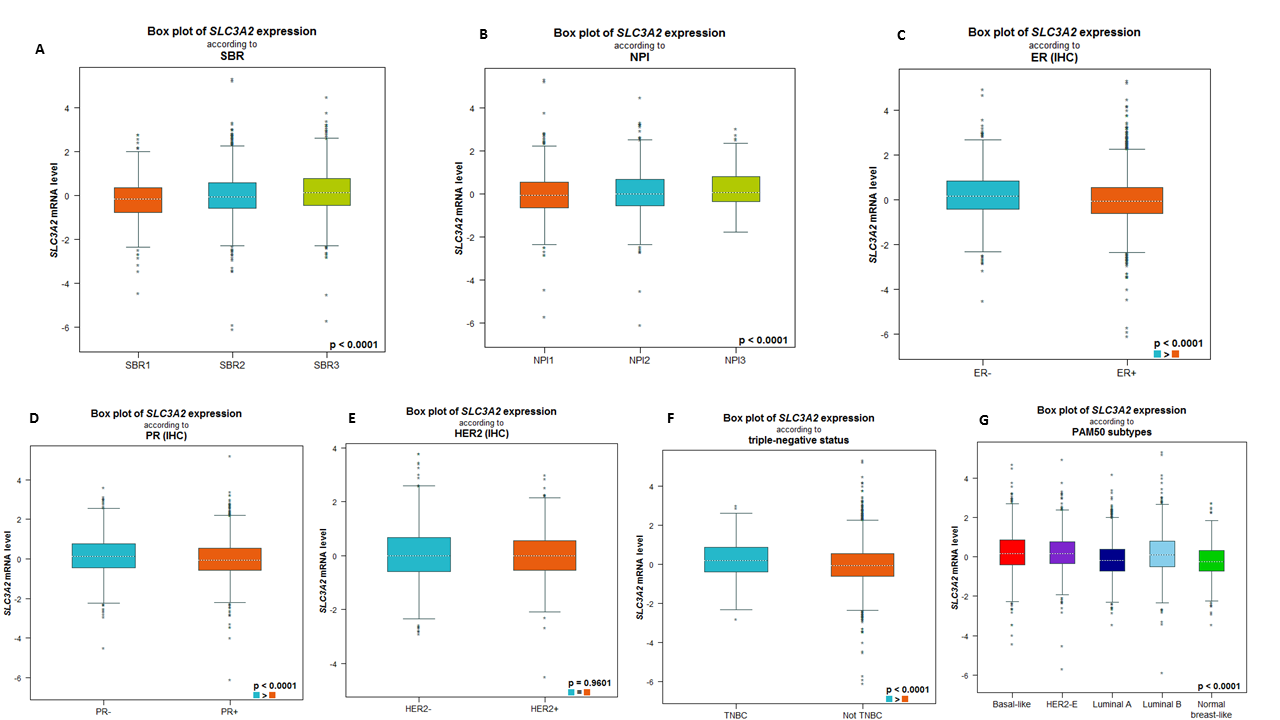


**Supplementary figure 1:** SLC3A2 gene expression and its association, using Breast Cancer Gene-Expression Miner, with: A) tumour grade, B) NPI, C) ER status, D) PR status, E) HER2 status, F) Triple Negative status, G) PAM50 subtypes.

**
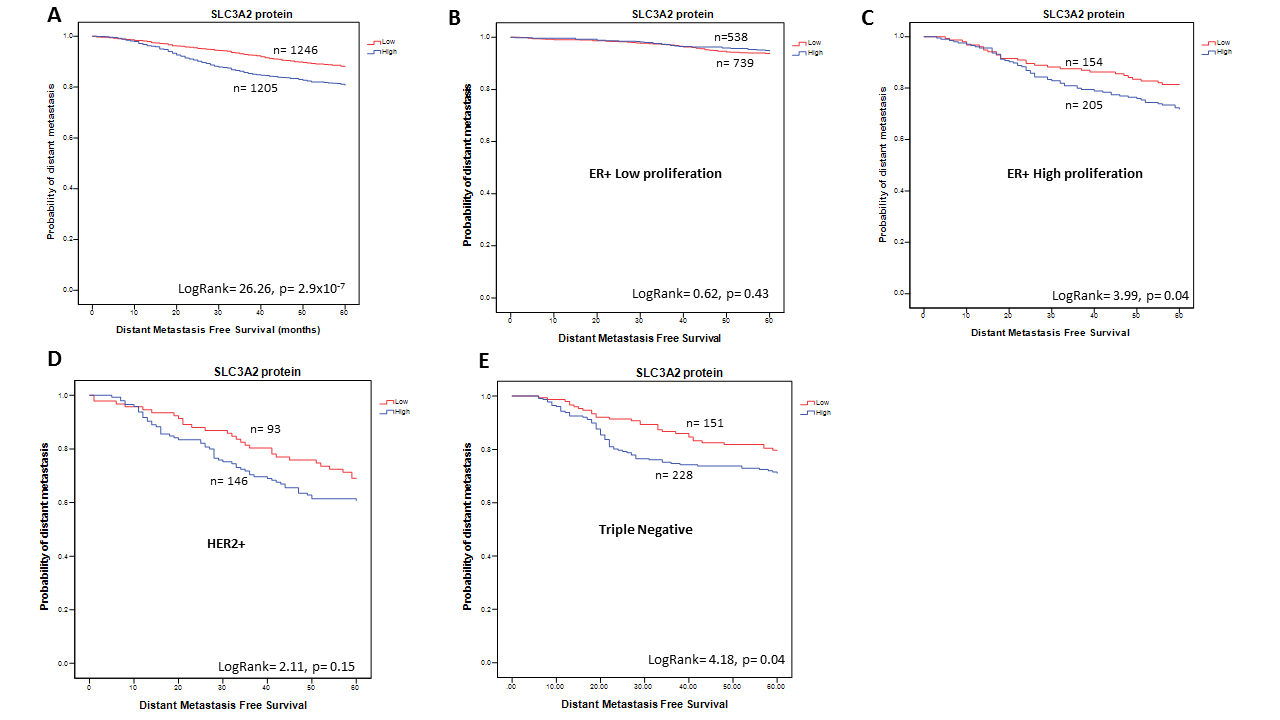
**

**Supplementary Figure 2.** SLC3A2 protein vs DMFS in A) all cases, B) ER+- Low Proliferation tumours, C) ER+- High Proliferation tumours, D) HER2+ tumours, E) Triple negative tumours.
